# Relationship between haemoglobin concentration and packed cell volume in cattle blood samples

**DOI:** 10.4102/ojvr.v82i1.863

**Published:** 2015-02-27

**Authors:** Paa-Kobina Turkson, Ebenezer Y. Ganyo

**Affiliations:** 1 Department of Animal Science, University of Cape Coast, Ghana; 2 Department of Biomedical and Forensic Sciences, University of Cape Coast, Ghana

## Abstract

A convention that has been adopted in medicine is to estimate haemoglobin (HB) concentration as a third of packed cell volume (PCV) or vice versa. The present research set out to determine whether a proportional relationship exists between PCV and Hb concentration in cattle blood samples, and to assess the validity of the convention of estimating Hb concentration as a third of PCV. A total of 440 cattle in Ghana from four breeds (Ndama, 110; West African Short Horn, 110; Zebu, 110 and Sanga, 110) were bled for haematological analysis, specifically packed cell volume, using the microhaematocrit technique and haemoglobin concentration using the cyanmethaemoglobin method. Means, standard deviations, standard errors of mean and 95% confidence intervals were calculated. Trendline analyses generated linear regression equations from scatterplots. For all the cattle, a significant and consistent relationship (*r* = 0.74) was found between Hb concentration and PCV (%). This was expressed as Hb concentration (g/dL) = 0.28 PCV + 3.11. When the Hb concentration was estimated by calculating it as a third of PCV, the relationship was expressed in linear regression as Hb concentration (g/dL) = 0.83 calculated Hb + 3.11. The difference in the means of determined (12.2 g/dL) and calculated (10.9 g/dL) Hb concentrations for all cattle was significant (*p* < 0.001), whereas the difference in the means of determined Hb and corrected calculated Hb was not significant. In conclusion, a simplified relationship of Hb (g/dL) = (0.3 PCV) + 3 may provide a better estimate of Hb concentration from the PCV of cattle.

## Introduction

Determining blood parameters is helpful in assessing the health status of animals. Common diseases in the tropics may lead to anaemia, examples of which include: helminthosis/helminthiasis, trypanosomosis, and tick-burden and tick-borne infections such as babesiosis and anaplasmosis. Measurement of anaemia is said to give a reliable indication of the disease status and production performance of trypanosome-infected animals (Nwoha & Anene 2011). Laboratory diagnosis of anaemia is based on the haemoglobin (Hb) concentration, the number of red blood cells and the haematocrit or packed cell volume (PCV) values (Aiello 1998). Anaemia is most simply and reliably estimated by measuring PCV percent using the haematocrit method, whilst determining the Hb concentration gives accurate information on the type of anaemia (Murray *et al.* 1983). Quinto *et al.* (2006) noted that measurement of haematocrit is easy and can be performed in most rural settings where methods of Hb concentration determination are unavailable, and rough estimates are made using observed PCV values, which is a much simpler and cheaper approach. In rural African human medicine clinical practices, haematocrit (PCV) values are commonly used because they are easy and cheaper to perform using manual techniques (Quinto *et al.* 2006). The same reason may hold true for use in veterinary practice. Haemoglobin concentration is measured using the cyanmethaemoglobin method, which is slightly more complex and more time consuming than the haematocrit method and is also less commonly used in laboratory investigation in animals (Murray *et al.* 1983).

A convention has been adopted in medical laboratory practice in estimating Hb concentration as a third of PCV or vice versa (Bain & Bates 2001). A similar conversion factor is used in veterinary laboratory practice (Jerry Oddoye [Accra Veterinary Investigation Laboratory] pers. comm., n.d.); however, there is hardly any information on the validity of this commonly used relationship between Hb concentration and PCV in veterinary medical practice.

The aims of the present study were: (1) to determine whether or not a proportional relationship exists between PCV and Hb concentration in cattle blood samples and (2) to assess the validity of the convention of estimating Hb concentration as a third of PCV. It was hoped that these would help to provide information that is relevant for fieldwork and clinical diagnosis.

## Materials and methods

A total of 440 cattle in Ghana from four breeds (Ndama, 110; West African Short Horn, 110; Zebu, 110 and Sanga, 110), that were randomly selected, were bled for haematological analysis as part of a larger study on trypanotolerance. Cattle less than 1 year old were classified as calves, those between 1 year and 3 years as young, and those older than 3 years were classified as adults.

### Packed cell volume determination

Packed cell volume, which is a measure of the proportion of the volume of the whole blood that is occupied by red blood cells, was determined by the microhaematocrit centrifugation technique (Jain 1986). Blood in a sample vacutainer tube was mixed by gently inverting the tube about 20 times. The blood was drawn three quarters of the way up a 75 mm x 1.0 mm microhaematocrit capillary tube. Blood was wiped off the tip of the capillary tube, and the end of the capillary tube was carefully plugged with plasticine. The capillary tube was placed, with the closed end outwards, in a microhaematocrit centrifuge (Hawksley & Sons Limited, England) and spun at 12 000 rpm for 5 min. The capillary tube was removed from the centrifuge, placed on a haematocrit reader and the PCV was recorded.

### Haemoglobin concentration determination

Haemoglobin concentration was measured spectro­photometrically by the cyanmethaemoglobin method (Jain 1986) by an experienced veterinary laboratory technologist in the National Veterinary Investigation Laboratory, Accra. Blood in a sample vacutainer tube was mixed gently by inverting about 20 times. Twenty microlitres of blood was added to 5 mL of Drabkin’s solution (containing potassium ferricyanide and potassium cyanide) in a test tube. In the Drabkin’s solution, the red blood cells were haemolysed and the haemoglobin was oxidised by the ferricyanide to methaemoglobin. The cyanide then converted the methaemoglobin to stable cyanmethaemoglobin. The mixture was allowed to stand for 15 min. After that, 1 mL of the mixture was pipetted into a cuvette. The cuvette was placed in a spectrophotometer (Jenway, England, Model: Genova MK3) set at 540 nm, and the absorbance of the cyanmethaemoglobin solution was read after zeroing the spectrophotometer using neutral Drabkin’s solution. The haemoglobin concentration of the blood sample was calculated by dividing the absorbance value by the slope obtained from a calibration graph. To obtain the calibration graph, a standard blood sample (of known haemoglobin concentration) was diluted with Drabkin’s solution: 5 in 0; 4 in 1; 3 in 2; 2 in 3 and 1 in 4. The absorbance of each of the five solutions was read in the spectrophotometer after the spectrophotometer was zeroed using neutral Drabkin’s solution. A graph of absorbance for each of the five solutions was plotted against the corresponding haemoglobin concentration, and the slope of the graph was determined. The haemoglobin concentration of each of the five solutions was obtained by multiplying the proportion of standard haemoglobin in that solution with the haemoglobin concentration value of the standard.

### Statistical analysis

Means, standard deviations (s.d.), standard errors (s.e.) of mean and 95% confidence intervals (95% CI) were calculated using standard formulae. Differences in the means of the determined and calculated Hb concentration values were tested using a two-tailed paired sample test in Microsoft Excel (Microsoft, USA).

Scatterplots were drawn using Microsoft Office Excel (version 2007, Microsoft, USA) matching determined Hb concentration with PCV, and determined Hb concentration with calculated Hb values (one third of PCV). Linear regression models were estimated to evaluate the relationship between PCV, determined Hb and calculated Hb values. Trendline analyses were used to generate the linear regression equations. To avoid the possibility of bias, separate regressions were performed on the basis of breed, age or sex. The significance of the correlation coefficient for the linear regression equations was tested using the formula suggested by Smillie (1966) and Varkevisser, Pathmanathan and Brownlee (1991) as follows:

$$t=r*(\sqrt{}((n-2)(1-r^{2}))$$ [Eqn 1]

where *t* = significance value; *r* = coefficient of correlation; *n* = number of samples.

Bland and Altman (1999) argued that since two methods designed to measure the same thing are bound to give a positive linear regression, the most useful comparison is to plot the difference between the measures against the mean of the two measures. This method was used when comparing determined Hb (g/dL) and calculated Hb (derived as PCV divided by a factor of three) before and after correction using the linear regression equation for all cattle. The differences between the determined and calculated Hb concentration values and the mean of the measurements [i.e. (determined Hb + calculated Hb)/2)] were calculated for each individual and used in a scatterplot. The plot of difference against mean allows for the investigation of any possible relationship between discrepancies and the true value (Bland & Altman 1999). The means and s.d. of the differences between Hb determined and calculated Hb values, on the one hand, and determined Hb and corrected calculated Hb concentration values, on the other hand, were calculated; 95% limits of agreement were computed as mean of difference ± 1.96 s.d. (Bland & Altman 1999). The 95% limits of agreement provided an interval within which 95% of the differences between measurements by the two methods were expected to lie (Bland & Altman 1999).

Spearman’s rank correlation coefficient (*r*_s_) was calculated, in Microsoft Excel, for the relationship between determined Hb and calculated Hb concentration, and also between the absolute differences and averages for individual samples, the latter as recommended by Bland and Altman (1999).

## Results

Table 1 presents haematological values (PCV, determined and calculated Hb concentration values) and linear regression parameters for determined Hb versus PCV, and determined Hb concentration versus calculated Hb for all the cattle, and also on the basis of breed, sex and age.

**TABLE 1:** Haematological and linear regression parameters according to breed, sex and category of cattle.

Variable	Class	*n*	PCV (%)		Hb Determined (g/dL)		Hb Calculated (g/dL)		Regression of Determined Hb versus PCV	Regression of Determined Hb versus calculated Hb	*r*	*t*-test value
			(x ± s.d.)	95% CI	(x ± s.d.)	95% CI	(x ± s.d.)	95% CI				
Breed	Wash	110	31.79 ± 5.60	30.74–32.84	11.79 ± 1.72	11.47–12.11	10.60 ± 1.58	10.30–10.90	*y* = 0.28; *x*_1_ + 3.00	*y* = 0.83; *x*_2_ + 3.00	0.76	12.22
	N’dama	110	34.50 ± 4.37	33.68–35.32	12.83 ± 1.67	12.52–13.14	11.50 ± 1.46	11.23–11.77	*y* = 0.27; *x*_1_ + 3.56	*y* = 0.81; *x*_2_ + 3.56	0.70	10.22
	Zebu	110	30.30 ± 4.95	29.37–31.23	11.58 ± 1.96	11.21–11.95	10.10 ± 1.65	9.79–10.41	*y* = 0.27; *x*_1_ + 3.52	*y* = 0.80; *x*_2_ + 3.52	0.67	9.45
	Sanga	110	34.39 ± 4.78	33.50–35.28	12.44 ± 1.88	12.09–12.79	11.46 ± 1.59	11.16–11.76	*y* = 0.30; *x*_1_ + 2.14	*y* = 0.90; *x*_2_ + 2.14	0.76	12.24
Sex	Male	174	32.17 ± 5.12	31.41–32.93	11.99 ± 1.92	11.70–12.28	10.72 ± 1.71	10.47–10.97	*y* = 0.27; *x*_1_ + 3.33	*y* = 0.81; *x*_2_ + 3.33	0.72	13.56
	Female	266	33.12 ± 4.93	32.53–33.71	12.26 ± 1.83	12.04–12.48	11.04 ± 1.64	10.84–11.24	*y* = 0.28; *x*_1_ + 2.96	*y* = 0.84; *x*_2_ + 2.96	0.76	18.73
Category	Calves	86	34.06 ± 5.76	32.84–35.28	12.42 ± 1.95	12.01–12.83	11.35 ± 1.92	10.94–11.76	*y* = 0.23; *x*_1_ + 4.43	*y* = 0.70; *x*_2_ + 4.43	0.69	8.86
	Young	138	33.41 ± 5.00	32.58–34.24	12.40 ± 1.94	12.08–12.72	11.14 ± 1.66	10.86–11.42	*y* = 0.28; *x*_1_ + 2.89	*y* = 0.85; *x*_2_ + 2.89	0.73	12.46
	Adult	216	31.80 ± 4.54	31.19–32.41	11.89 ± 1.76	11.66–12.12	10.60 ± 1.51	10.40–10.80	*y* = 0.30; *x*_1_+ 2.43	*y* = 0.89; *x*_2_ + 2.43	0.77	17.53
**Total**	**All**	**440**	**32.75 ± 5.02**	**32.28–33.22**	**12.15 ± 1.87**	**11.98–12.32**	**10.92 ± 1.67**	**10.76–11.08**	***y* = 0.28; *x*_1_ + 3.11**	***y* = 0.83; *x*_2_ + 3.11**	**0.74**	**23.16**

PCV, packed cell volume; s.d., standard deviation; CI, confidence interval; Hb, haemoglobin concentration; *y*, determined Hb; *x*_1_, PCV; *x*_2_, calculated Hb (i.e. PCV/3); g/dL, grams per decilitre; *r*, correlation coefficient.

The proportion of samples for which determined Hb concentration was higher than calculated Hb concentration was 86.1% (379/440) and was significant (*p* < 0.05). After correction using the regression equation for all cattle, the proportion of samples for which determined Hb concentration was higher than calculated Hb concentration dropped to 46.4%. The difference in the means of determined (12.2 g/dL) and calculated (10.9 g/dL) Hb concentrations for all cattle was significant (*p* < 0.001), whereas the difference in the means of determined Hb and corrected calculated Hb concentrations was not significant (*p* > 0.05).

The mean (± s.d.) of differences between determined and calculated Hb concentration values was 1.24 ± 1.29 (range: -3.56–7.25; lower 95% limit of agreement was -1.28; upper 95% limit of agreement was 3.76). The mean (± s.d.) of differences between determined and corrected calculated Hb concentration values was 0.00 ± 1.25 (range: -4.61–5.63; lower 95% limit of agreement -2.46; upper 95% limit of agreement 2.46).

Spearman’s rank correlation coefficient for the relationship between determined Hb and calculated Hb concentrations, (*r_s_* = 0.73) was significantly different from one (*p* < 0.001, degrees of freedom [*df*] = 438), whilst that for the relationship between absolute differences and averages of determined and calculated Hb concentration (*r_s_* = 0.18) was barely significant at 5% significance level, but not at 1%.

**FIGURE 1:**
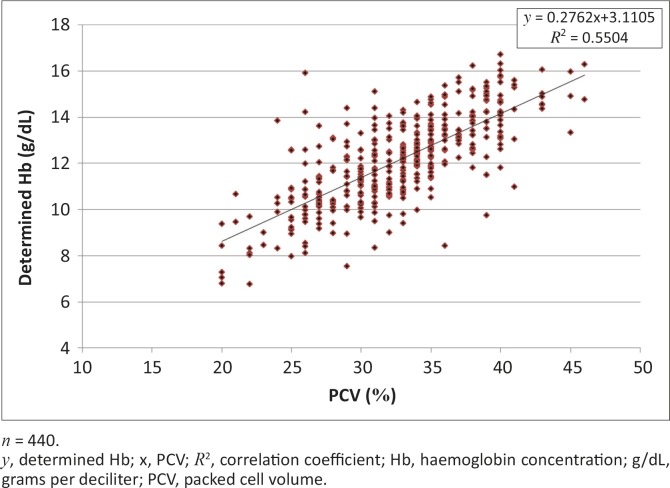
Scatterplot of determined haemoglobin concentration versus packed cell volume for all cattle.

**FIGURE 2:**
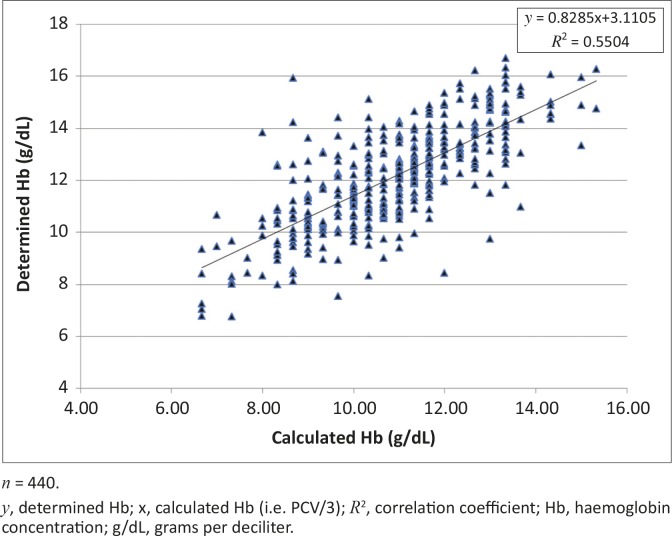
Scatterplot of determined versus calculated haemoglobin concentrations for all cattle.

**FIGURE 3:**
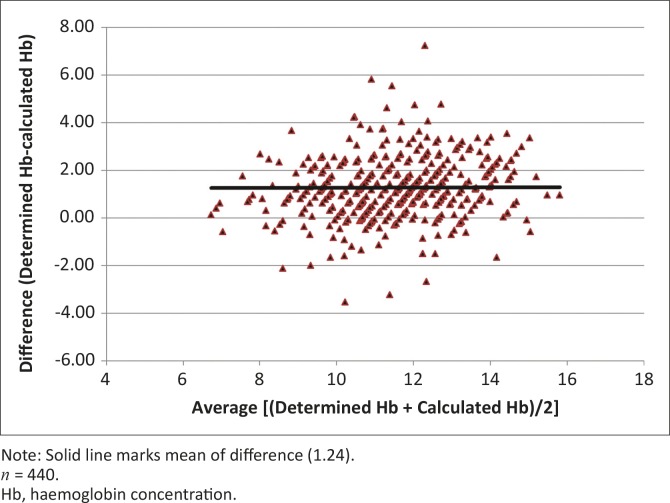
Scatterplot of difference against average of determined haemoglobin and calculated haemoglobin concentrations for all cattle.

**FIGURE 4:**
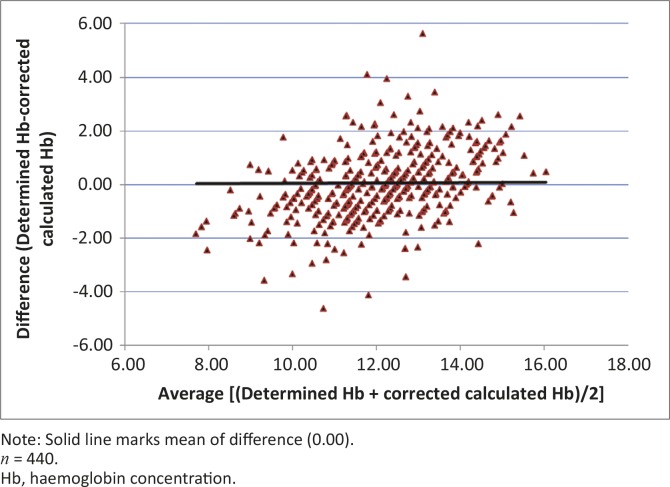
Scatterplot of difference after correction against average of determined haemoglobin and corrected calculated haemoglobin concentrations for all cattle.

Figures 1 and 2 show scatterplots of the determined Hb concentration versus PCV and the determined Hb versus calculated Hb concentrations, respectively. Figures 3 and 4 show scatterplots of the differences versus averages for the determined and calculated Hb concentrations (with a solid line marking the mean of 1.24) and differences versus averages for the determined and corrected calculated Hb concentration (with a solid line marking the mean of 0.0), respectively.

## Discussion

For all of the cattle, a significant and consistent relationship was found between Hb concentration and PCV (%). This was expressed as Hb concentration (g/dL) = 0.28 PCV + 3.11. When the Hb concentration was estimated as a third of PCV, the relationship was expressed as Hb concentration (g/dL) = 0.83 calculated Hb + 3.11 (Table 1). The findings are indicative of the potential magnitude of the problem of using the convention of estimating Hb concentrations (g/dL) as a third of PCV values (%). The results show a consistent and significant bias (underestimation) of calculated Hb concentration compared to measured Hb (determined Hb in the present study) (Table 1). The differences between the means were also significantly different (*p* < 0.05) and cut across breed, sex and age categories (Table 1).

In a study on humans, Carneiro *et al.* (2007) found that Hb concentration measurements were lower than the values obtained from PCV/3. In contrast, the present study found that 86% of the Hb concentration values obtained by the cyanmethaemoglobin method were higher than the Hb concentrations estimated as a third of PCV. In effect, whereas in their study there was a likelihood of overestimation, in the present study the result was underestimation, which could affect clinical treatment plans.

Linear regression analysis was employed to determine whether the relationship between PCV and Hb concentration differed on the basis of breed, sex and age categories of the cattle. The individual slopes within breed, sex and age categories did not differ significantly, except possibly for calves. These results may imply that a simplified relationship of Hb (g/dL) = 0.3 PCV + 3 may provide a more reasonable and better estimate of Hb concentration from the PCV of cattle. In cattle, the convention of estimating the Hb concentration as a third of PCV would need modifying to be a third of PCV + 3.

The convention or standard of estimating Hb has been used extensively in medicine to estimate the prevalence of anaemia (Carneiro *et al.* 2007; Quinto *et al.* 2006; Rodriquez-Morales *et al.* 2007; World Health Organization [WHO] 1968). Recently, the convention was recommended for birds in eight orders (Velguth, Payton & Hoover 2010). Quinto *et al.* (2006) noted that although Hb and haematocrit (PCV) are closely related, the usual transformation of three times the haemoglobin (g/dL) equals the PCV is inaccurate. The present findings support the reports from studies in human medicine that Hb concentration levels could not be derived from PCV values with an acceptable accuracy using the general rule of dividing by three (Carneiro *et al.* 2007; Quinto *et al.* 2006; Rodriquez-Morales *et al.* 2007). These studies also showed that the relationship between Hb concentration and PCV was not exactly three and could be affected by factors such as sex and age in humans.

The relationship between PCV and Hb is expressed in the Mean Corpuscular Haemoglobin Concentration (MCHC) (Quinto *et al.* 2006). This is an indicator of the concentration of Hb per unit volume of red blood cell expressed in g/dL as [(Hb X 100)/PCV] and is more accurate than mean corpuscular haemoglobin (MCH) (Aiello 1998). Therefore, any estimation of Hb concentration from PCV that is unreliable or invalid would affect the calculation of MCHC so that clinical decisions made on the basis of MCHC may not be appropriate.

In conclusion, a simplified relationship of Hb (g/dL) = (0.3 PCV) + 3 may provide a better estimate of Hb concentration from the PCV of cattle. It is, therefore, recommended that if it is necessary to estimate Hb concentration from PCV value, then the simplified relationship may be more appropriate to use.
